# Poly[bis­(μ_6_-benzene-1,3,5-tricarboxyl­ato-κ^7^ *O* ^1^,*O* ^1′^:*O* ^1′^:*O* ^3^:*O* ^3′^:*O* ^5^:*O* ^5′^)tetra­kis­(dimethyl­formamide-κ*O*)trimagnesium(II)]

**DOI:** 10.1107/S1600536812023240

**Published:** 2012-05-31

**Authors:** Katarzyna Łuczyńska-Szymczak, Wojciech Starosta, Janusz Leciejewicz

**Affiliations:** a Institute of Nuclear Chemistry and Technology, ul. Dorodna 16, 03-195 Warszawa, Poland

## Abstract

The asymmetric unit of the polymeric title compound, [Mg_3_(C_9_H_3_O_6_)_2_(C_3_H_7_NO)_4_]_*n*_, contains three Mg^II^ ions bridged by carboxyl­ate O atoms from two fully deprotonated benzene-1,3,5-tricarboxyl­ate (BTC) trianions and four metal-coordinated dimethyl­formamide (DMF) mol­ecules. One Mg^II^ ion is octa­hedrally coordinated by six carboxyl­ate O atoms. The other two cations are each octa­hedrally coordinated by four carboxyl­ate O atoms and two O atoms donated by two DMF mol­ecules: in one, the DMF mol­ecules are *cis* and in the other they are *trans*. The three Mg^II^ octa­hedra form clusters, which are bridged by the BTC trianions, generating a three-dimensional structure.

## Related literature

For the crystal structures of four Mg^II^ complexes with benzene-1,3,5- tricarboxyl­ate ligands, see: Davies *et al.* (2007 ▶); Ma *et al.* (2007 ▶); Song *et al.* (2010 ▶); Yeh *et al.* (2010 ▶).
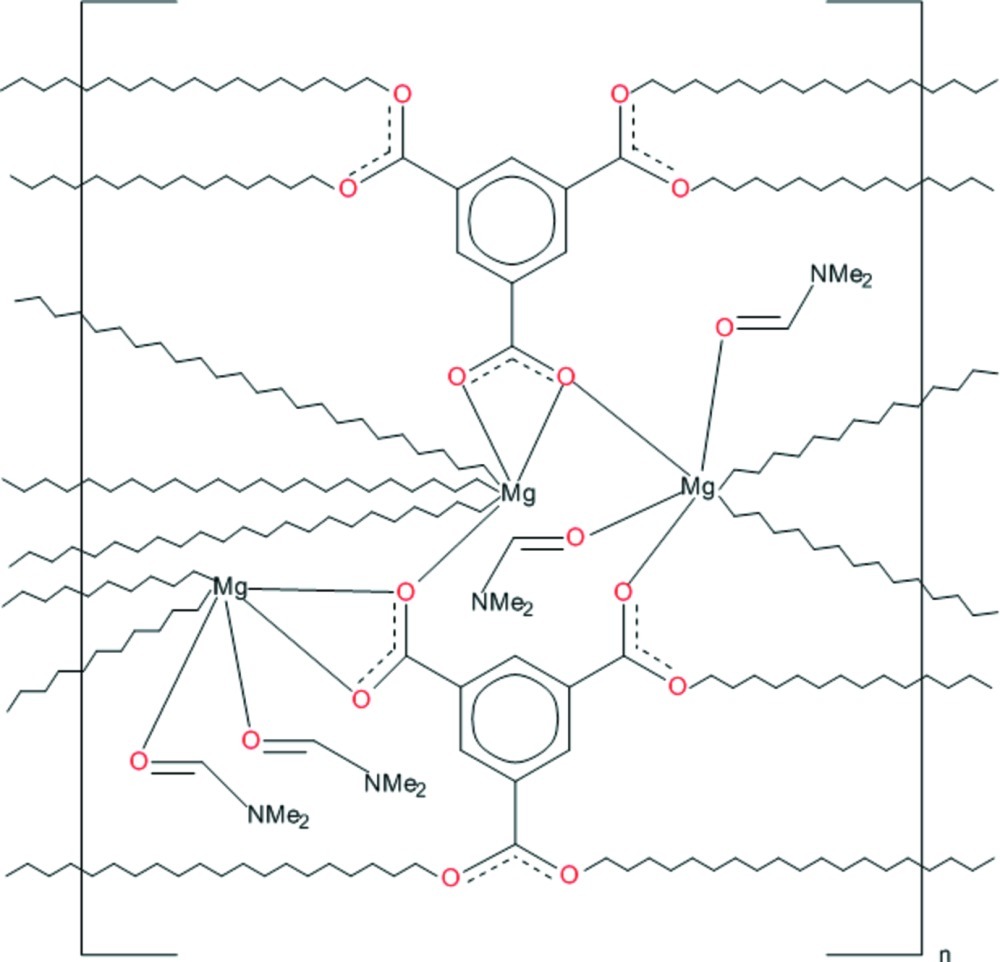


## Experimental

### 

#### Crystal data

[Mg_3_(C_9_H_3_O_6_)_2_(C_3_H_7_NO)_4_]
*M* *_r_* = 779.54
Monoclinic, 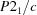

*a* = 17.566 (4) Å
*b* = 11.961 (2) Å
*c* = 18.514 (4) Å
β = 116.65 (3)°
*V* = 3476.9 (12) Å^3^
*Z* = 4
Mo *K*α radiation
μ = 0.17 mm^−1^
*T* = 293 K
0.14 × 0.13 × 0.07 mm

#### Data collection

Kuma KM-4 four-cricle diffractometer
Absorption correction: analytical (*CrysAlis RED*; Oxford Diffraction, 2008 ▶) *T*_min_ = 0.979, *T*_max_ = 0.991
5218 measured reflections
4986 independent reflections
2425 reflections with *I* > 2σ(*I*)
*R*_int_ = 0.059
3 standard reflections every 200 reflections intensity decay: 4.2%

#### Refinement

*R*[*F* ^2^ > 2σ(*F* ^2^)] = 0.041
*wR*(*F* ^2^) = 0.144
*S* = 1.00
4986 reflections
486 parameters
H-atom parameters constrained
Δρ_max_ = 0.48 e Å^−3^
Δρ_min_ = −0.38 e Å^−3^

### 

Data collection: *KM-4 Software* (Kuma, 1996 ▶); cell refinement: *KM-4 Software*; data reduction: *DATAPROC* (Kuma, 2001 ▶); program(s) used to solve structure: *SHELXS97* (Sheldrick, 2008 ▶); program(s) used to refine structure: *SHELXL97* (Sheldrick, 2008 ▶); molecular graphics: *SHELXTL* (Sheldrick, 2008 ▶); software used to prepare material for publication: *SHELXTL*.

## Supplementary Material

Crystal structure: contains datablock(s) I, global. DOI: 10.1107/S1600536812023240/hb6785sup1.cif

Structure factors: contains datablock(s) I. DOI: 10.1107/S1600536812023240/hb6785Isup2.hkl

Additional supplementary materials:  crystallographic information; 3D view; checkCIF report

## Figures and Tables

**Table 1:** Selected bond lengths (Å)

Mg1—O14^i^	1.997 (3)
Mg1—O24^ii^	2.041 (3)
Mg1—O15^iii^	2.072 (3)
Mg1—O12	2.169 (3)
Mg1—O11	2.202 (3)
Mg1—O21	2.233 (3)
Mg2—O23^ii^	2.019 (3)
Mg2—O16^iii^	2.023 (4)
Mg2—O12	2.058 (3)
Mg2—O41	2.081 (4)
Mg2—O31	2.091 (4)
Mg2—O26	2.113 (4)
Mg3—O21	2.279 (3)
Mg3—O22	2.104 (4)
Mg3—O13^i^	1.990 (3)
Mg3—O25^i^	2.024 (3)
Mg3—O51	2.089 (4)
Mg3—O61	2.110 (5)

Symmetry codes: (i) 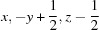; (ii) 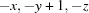; (iii) 
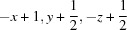
.

**Fig. 1.**
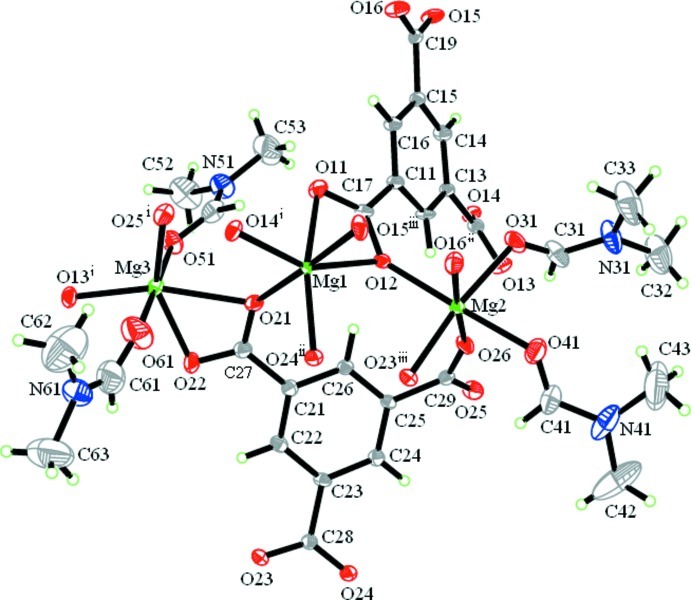
The asymmetric unit of the title compound (50% probability displacement ellipsoids) expanded to show the complete metal coordination spheres. Symmetry code: ix, -y + 1/2, z - 1/2; ii -x, -y + 1/2, -z; iii -x + 1, y + 1/2, -z + 1/2.

**Fig. 2.**
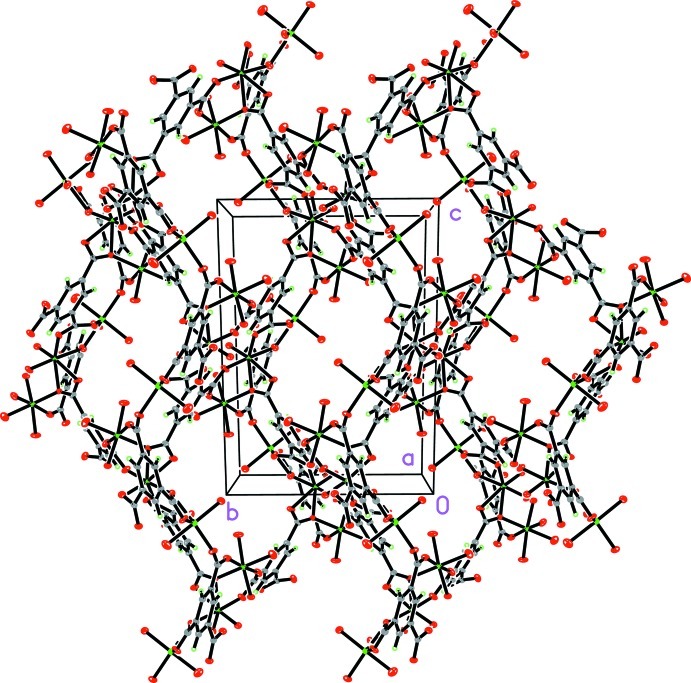
Packing diagram of the structure viewed along the a axis.

## supplementary crystallographic information

### Comment

Magnesium(II) coordination polymers have been recently attracting considerable interest as possible metal-organic frameworks (MOF) with expected potential practical applications. Crystal structures of four Mg^II^ coordination polymers with benzene-1,3,5-tricarboxylate (BTC) ligand have been up to now reported (Ma *et al.*, 2007; Davies *et al.*, 2007; Yeh *et al.*, 2010). Continuing our interest in Mg^II^ coordination chemistry we have synthesized a new Mg^II^ complex with BTC ligand. Its structure is reported below. The asymmetric cell of the title compound contains three symmetry independent Mg^II^ ions, each with a distorted octahedral coordination geometry, two symmetry independent fully deprotonated BTC ligand molecules and four symmetry independent dimethylformamide (DMF) molecules. While the distorted octahedral coordination of the Mg1 ion is composed of six carboxylato O atoms donated by both BTC ligands, the coordination environment of the Mg2 and Mg3 ions is built each of four ligand carboxylate O atoms and two DMF O atoms. The Mg—O bond distances collected in Table 1 do not differ from those reported in in other Mg^II^ complexes with the title ligand. Both BTC ligand molecules show µ_6_ bridging mode (Fig.1). Carboxylate O11 and O12 atoms of the BTC1 ligand coordinate the Mg1 ion, but the O12 atom acts as bidentate and is bonded also to the Mg2 ion. The carboxylate group with O13 and O14 atoms bridges the Mg3^iv^ and Mg1^iv^ ions; the carboxylate group with O15 and O16 atoms bridges the Mg1^v^ and Mg2^v^ ions. Ligand molecule BTC2 uses its carboxylate group with O21 and O22 atoms to bridge Mg1 and Mg3 ions; the carboxylate group with O23 and O24 atoms bridges the Mg2^ii^ and Mg1^ii^ion, while the carboxylate group with O25 and O26 atoms bridges the Mg3^iv^ and Mg2 ions. (Symmetry code: ^ii^ -*x*, -*y* + 1, -*z*; ^iv^*x*, -*y* + 1/2, *z* + 1/2; ^v^ -*x* + 1, *y* - 1/2, -*z* + 1/2). BTC1 and BTC2 benzene rings are planar with r.m.s of 0.0192 (2) Å and 0.0106 (2) Å, respectively. The carboxylate groups C17/O11/O12, C18/O13/O14 and C19/O15/O16 deviate from the benzene ring 1 by 29.1 (5)°, 35.1 (5)° and 9.5 (5)°, respectively. The respective values for benzene ring 2 and carboxylate groups C27/O21/O22, C28/O23/O24 and C29/O25/O26 are 21.2 (5)°, 9.3 (5)° and 42.3 (5)°. Four DMF molecules show r.m.s. values between 0.0095 (1) Å and 0.0134 (1) Å. Three Mg^II^ octahedra linked along Mg3—O21—Mg1—O12—Mg2 bonding pathway clearly visible in Fig.1. form clusters which, bridged by BTC carboxylate O atoms, give rise to channels propagating along crystal *a* direction with DMF molecules localized inside (Fig.2). For comparison, a two-dimensional structure of a trigonal complex in which the BTC ligand acts also in µ_6_ and the formate ligand in µ_3_ modes bridge three symmetry independent Mg^II^ ions has been recently reported (Yeh *et al.*, 2010). In the rhombohedral layer structure of a complex with BTC and DMA (dimethyloacetamide) ligands, two BTC molecules, each showing µ_3_ bridging mode are observed. Mg^II^ ions and BTC molecules are nearly coplanar and four carboxylate O atoms form an equatorial plane of a distorted octahedral environment around a Mg^II^ ion with O atoms donated by two DMA molecules at apical positions (Davies *et al.*, 2007). A similar structure but with DMF O atoms at axial positions is also known (Song *et al.*, 2010). In an orthorhombic Mg^II^ complex with BTC ligand and DMA a three-dimensional framework with pores filled by the DMA molecules has been reported (Ma *et al.*, 2007).

### Experimental

0.25 g of magnesium nitrate hexahydrate and 0.14 g of benzene-1,3,5-tricarboxylic acid were dissolved with stirring for two hours in 17 ml of DMF, inserted into a Parr autoclave and heated for three days at 400 K, than cooled to room temperatute. Colourless blocks were extracted from the reaction vessel, washed with cold ethanol and dried in the air.

### Refinement

H atoms attached to benzene-ring and methyl groups C atoms were located at calculated positions and treated as riding on the parent atoms with C—H=0.93 Å and *U*_iso_(H)=1.2*U*_eq_(C).

### Crystal data

**Table:**

[Mg_3_(C_9_H_3_O_6_)_2_(C_3_H_7_NO)_4_]	*F*(000) = 1624
*M**_r_* = 779.54	*D*_x_ = 1.489 Mg m^−^^3^
Monoclinic, *P*2_1_/*c*	Mo *K*α radiation, λ = 0.71073 Å
Hall symbol: -P 2ybc	Cell parameters from 25 reflections
*a* = 17.566 (4) Å	θ = 6–15°
*b* = 11.961 (2) Å	µ = 0.17 mm^−^^1^
*c* = 18.514 (4) Å	*T* = 293 K
β = 116.65 (3)°	Block, colourless
*V* = 3476.9 (12) Å^3^	0.14 × 0.13 × 0.07 mm
*Z* = 4	

### Data collection

**Table:**

Kuma KM-4 four-cricle diffractometer	2425 reflections with *I* > 2σ(*I*)
Radiation source: fine-focus sealed tube	*R*_int_ = 0.059
Graphite monochromator	θ_max_ = 24.2°, θ_min_ = 1.3°
profile data from ω/2θ scans	*h* = −17→18
Absorption correction: analytical (*CrysAlis RED*; Oxford Diffraction, 2008)	*k* = −13→0
*T*_min_ = 0.979, *T*_max_ = 0.991	*l* = −19→0
5218 measured reflections	3 standard reflections every 200 reflections
4986 independent reflections	intensity decay: 4.2%

### Refinement

**Table:**

Refinement on *F*^2^	Primary atom site location: structure-invariant direct methods
Least-squares matrix: full	Secondary atom site location: difference Fourier map
*R*[*F*^2^ > 2σ(*F*^2^)] = 0.041	Hydrogen site location: inferred from neighbouring sites
*wR*(*F*^2^) = 0.144	H-atom parameters constrained
*S* = 1.00	*w* = 1/[σ^2^(*F*_o_^2^) + (0.0748*P*)^2^ + 2.4811*P*] where *P* = (*F*_o_^2^ + 2*F*_c_^2^)/3
4986 reflections	(Δ/σ)_max_ < 0.001
486 parameters	Δρ_max_ = 0.48 e Å^−^^3^
0 restraints	Δρ_min_ = −0.38 e Å^−^^3^

### Special details

**Table:**

Geometry. All e.s.d.'s (except the e.s.d. in the dihedral angle between two l.s. planes) are estimated using the full covariance matrix. The cell e.s.d.'s are taken into account individually in the estimation of e.s.d.'s in distances, angles and torsion angles; correlations between e.s.d.'s in cell parameters are only used when they are defined by crystal symmetry. An approximate (isotropic) treatment of cell e.s.d.'s is used for estimating e.s.d.'s involving l.s. planes.
Refinement. Refinement of *F*^2^ against ALL reflections. The weighted *R*-factor *wR* and goodness of fit *S* are based on *F*^2^, conventional *R*-factors *R* are based on *F*, with *F* set to zero for negative *F*^2^. The threshold expression of *F*^2^ > σ(*F*^2^) is used only for calculating *R*-factors(gt) *etc*. and is not relevant to the choice of reflections for refinement. *R*-factors based on *F*^2^ are statistically about twice as large as those based on *F*, and *R*- factors based on ALL data will be even larger.

### Fractional atomic coordinates and isotropic or equivalent isotropic displacement parameters (Å^2^)

**Table:**

	*x*	*y*	*z*	*U*_iso_*/*U*_eq_	
Mg1	0.28724 (9)	0.41852 (12)	0.02746 (8)	0.0115 (3)	
Mg2	0.28781 (9)	0.55240 (12)	0.19953 (9)	0.0179 (4)	
O12	0.30491 (18)	0.4054 (2)	0.15088 (17)	0.0165 (7)	
O14	0.32096 (19)	0.1175 (3)	0.44045 (18)	0.0211 (7)	
O11	0.3730 (2)	0.2903 (3)	0.10858 (18)	0.0254 (8)	
O15	0.61469 (19)	0.0343 (3)	0.43342 (19)	0.0269 (8)	
O13	0.2857 (2)	0.2948 (3)	0.4019 (2)	0.0262 (8)	
O16	0.6279 (2)	0.1229 (3)	0.3335 (2)	0.0281 (8)	
C13	0.3768 (3)	0.2027 (4)	0.3594 (3)	0.0164 (10)	
C12	0.3488 (3)	0.2661 (4)	0.2897 (3)	0.0193 (11)	
H12	0.2997	0.3090	0.2735	0.023*	
C11	0.3932 (3)	0.2664 (4)	0.2437 (3)	0.0185 (11)	
C14	0.4531 (3)	0.1429 (4)	0.3860 (3)	0.0214 (11)	
H14	0.4714	0.0984	0.4319	0.026*	
C17	0.3569 (3)	0.3244 (4)	0.1638 (3)	0.0177 (10)	
C18	0.3242 (3)	0.2051 (4)	0.4049 (3)	0.0185 (10)	
C16	0.4697 (3)	0.2101 (4)	0.2722 (3)	0.0179 (10)	
H16	0.5007	0.2125	0.2426	0.022*	
C15	0.5018 (3)	0.1497 (4)	0.3440 (3)	0.0183 (11)	
C19	0.5879 (3)	0.0977 (4)	0.3725 (3)	0.0185 (10)	
O31	0.3879 (2)	0.5254 (3)	0.3146 (2)	0.0348 (9)	
O41	0.2760 (2)	0.7044 (3)	0.2482 (2)	0.0385 (9)	
C41	0.2128 (4)	0.7361 (5)	0.2531 (4)	0.0461 (16)	
H41	0.1617	0.7014	0.2193	0.055*	
N41	0.2095 (4)	0.8147 (4)	0.3013 (4)	0.0668 (17)	
C42	0.1286 (7)	0.8435 (8)	0.3020 (7)	0.115 (4)	
H42A	0.0822	0.8265	0.2503	0.173*	
H42B	0.1280	0.9219	0.3130	0.173*	
H42C	0.1227	0.8010	0.3432	0.173*	
O21	0.18676 (19)	0.2866 (3)	−0.00975 (18)	0.0228 (8)	
N31	0.4322 (3)	0.6032 (5)	0.4372 (3)	0.0497 (14)	
C31	0.3803 (3)	0.5400 (5)	0.3764 (3)	0.0407 (15)	
H31	0.3355	0.5046	0.3809	0.049*	
C32	0.4186 (5)	0.6223 (7)	0.5086 (4)	0.077 (2)	
H32A	0.4714	0.6126	0.5564	0.115*	
H32B	0.3774	0.5699	0.5090	0.115*	
H32C	0.3981	0.6970	0.5072	0.115*	
C33	0.5010 (5)	0.6656 (7)	0.4322 (4)	0.081 (3)	
H33A	0.5517	0.6590	0.4824	0.121*	
H33B	0.4852	0.7429	0.4217	0.121*	
H33C	0.5113	0.6359	0.3893	0.121*	
O22	0.0627 (2)	0.2178 (3)	−0.09433 (19)	0.0311 (9)	
C27	0.1073 (3)	0.2737 (4)	−0.0321 (3)	0.0223 (11)	
C26	0.1161 (3)	0.3541 (4)	0.0967 (3)	0.0175 (10)	
H26	0.1746	0.3426	0.1204	0.021*	
C21	0.0661 (3)	0.3227 (4)	0.0163 (3)	0.0189 (11)	
C25	0.0790 (3)	0.4025 (4)	0.1413 (3)	0.0156 (10)	
Mg3	0.16582 (10)	0.17878 (13)	−0.11854 (9)	0.0197 (4)	
O51	0.2005 (2)	0.0431 (3)	−0.0391 (2)	0.0326 (9)	
O61	0.1369 (3)	0.3167 (4)	−0.1975 (3)	0.0616 (13)	
N51	0.2772 (3)	−0.0425 (4)	0.0807 (3)	0.0405 (12)	
N61	0.0720 (3)	0.4035 (4)	−0.3183 (3)	0.0512 (14)	
C51	0.2573 (4)	0.0438 (4)	0.0312 (3)	0.0335 (13)	
H51	0.2883	0.1094	0.0504	0.040*	
C52	0.2318 (5)	−0.1473 (5)	0.0551 (4)	0.069 (2)	
H52A	0.1762	−0.1397	0.0526	0.103*	
H52B	0.2627	−0.2049	0.0931	0.103*	
H52C	0.2262	−0.1668	0.0027	0.103*	
C61	0.0773 (5)	0.3476 (6)	−0.2541 (5)	0.070 (2)	
H61	0.0254	0.3307	−0.2546	0.084*	
C22	−0.0204 (3)	0.3412 (4)	−0.0185 (3)	0.0197 (11)	
H22	−0.0540	0.3180	−0.0713	0.024*	
C53	0.3467 (4)	−0.0391 (6)	0.1617 (4)	0.071 (2)	
H53A	0.3877	−0.0957	0.1671	0.107*	
H53B	0.3250	−0.0525	0.2002	0.107*	
H53C	0.3733	0.0331	0.1714	0.107*	
C28	−0.1514 (3)	0.4268 (4)	−0.0213 (3)	0.0173 (10)	
C24	−0.0081 (3)	0.4222 (4)	0.1043 (3)	0.0189 (11)	
H24	−0.0331	0.4551	0.1339	0.023*	
C23	−0.0586 (3)	0.3941 (4)	0.0241 (3)	0.0181 (11)	
C62	0.1435 (6)	0.4292 (8)	−0.3309 (6)	0.111 (3)	
H62A	0.1945	0.4062	−0.2847	0.167*	
H62B	0.1455	0.5084	−0.3385	0.167*	
H62C	0.1393	0.3907	−0.3780	0.167*	
C63	−0.0109 (5)	0.4319 (8)	−0.3775 (6)	0.125 (4)	
H63A	−0.0494	0.4311	−0.3537	0.187*	
H63B	−0.0294	0.3786	−0.4209	0.187*	
H63C	−0.0100	0.5052	−0.3982	0.187*	
O23	−0.18677 (18)	0.4056 (3)	−0.09475 (18)	0.0226 (8)	
O24	−0.18513 (19)	0.4760 (3)	0.01737 (18)	0.0213 (8)	
O26	0.20657 (19)	0.4791 (3)	0.24270 (18)	0.0238 (8)	
C29	0.1363 (3)	0.4358 (4)	0.2277 (3)	0.0200 (11)	
O25	0.1109 (2)	0.4163 (3)	0.28056 (19)	0.0263 (8)	
C43	0.2881 (6)	0.8664 (8)	0.3590 (5)	0.115 (4)	
H43A	0.3028	0.8380	0.4122	0.172*	
H43B	0.2806	0.9459	0.3585	0.172*	
H43C	0.3330	0.8494	0.3447	0.172*	

### Atomic displacement parameters (Å^2^)

**Table:**

	*U*^11^	*U*^22^	*U*^33^	*U*^12^	*U*^13^	*U*^23^
Mg1	0.0103 (7)	0.0163 (8)	0.0089 (7)	0.0006 (6)	0.0054 (6)	−0.0010 (6)
Mg2	0.0159 (8)	0.0232 (9)	0.0145 (8)	−0.0007 (6)	0.0066 (6)	−0.0033 (7)
O12	0.0150 (16)	0.0188 (17)	0.0136 (16)	0.0035 (14)	0.0046 (13)	0.0031 (13)
O14	0.0250 (18)	0.0208 (19)	0.0240 (18)	0.0033 (14)	0.0168 (14)	0.0058 (14)
O11	0.029 (2)	0.036 (2)	0.0148 (18)	0.0111 (16)	0.0131 (15)	0.0050 (15)
O15	0.025 (2)	0.036 (2)	0.024 (2)	0.0174 (15)	0.0145 (15)	0.0142 (16)
O13	0.0266 (19)	0.025 (2)	0.035 (2)	0.0093 (15)	0.0212 (16)	0.0072 (15)
O16	0.024 (2)	0.035 (2)	0.032 (2)	0.0097 (15)	0.0180 (16)	0.0133 (16)
C13	0.015 (2)	0.020 (3)	0.013 (2)	0.004 (2)	0.0055 (19)	0.0034 (19)
C12	0.010 (2)	0.025 (3)	0.025 (3)	0.005 (2)	0.010 (2)	0.005 (2)
C11	0.014 (2)	0.022 (3)	0.019 (3)	0.002 (2)	0.007 (2)	0.002 (2)
C14	0.020 (3)	0.025 (3)	0.020 (3)	0.003 (2)	0.009 (2)	0.006 (2)
C17	0.011 (2)	0.022 (3)	0.020 (3)	0.003 (2)	0.0065 (19)	0.005 (2)
C18	0.017 (2)	0.022 (3)	0.016 (3)	0.002 (2)	0.007 (2)	0.003 (2)
C16	0.016 (2)	0.027 (3)	0.015 (3)	0.005 (2)	0.010 (2)	0.006 (2)
C15	0.016 (2)	0.025 (3)	0.015 (3)	0.003 (2)	0.0083 (19)	0.001 (2)
C19	0.018 (3)	0.023 (3)	0.015 (3)	0.004 (2)	0.008 (2)	−0.002 (2)
O31	0.026 (2)	0.058 (3)	0.014 (2)	0.0034 (17)	0.0037 (15)	0.0009 (17)
O41	0.041 (2)	0.039 (2)	0.042 (2)	−0.0016 (19)	0.0242 (18)	−0.0121 (18)
C41	0.058 (4)	0.035 (4)	0.048 (4)	−0.003 (3)	0.027 (3)	−0.015 (3)
N41	0.107 (5)	0.049 (3)	0.067 (4)	0.019 (3)	0.059 (4)	−0.006 (3)
C42	0.156 (9)	0.088 (7)	0.176 (10)	0.049 (6)	0.139 (9)	0.021 (7)
O21	0.0158 (19)	0.0274 (19)	0.0264 (19)	−0.0006 (14)	0.0105 (14)	−0.0057 (15)
N31	0.048 (3)	0.079 (4)	0.027 (3)	−0.033 (3)	0.021 (2)	−0.018 (3)
C31	0.031 (3)	0.056 (4)	0.028 (3)	−0.017 (3)	0.007 (3)	0.001 (3)
C32	0.081 (5)	0.114 (7)	0.040 (4)	−0.032 (5)	0.032 (4)	−0.017 (4)
C33	0.076 (5)	0.116 (7)	0.058 (5)	−0.065 (5)	0.038 (4)	−0.030 (5)
O22	0.0224 (19)	0.040 (2)	0.029 (2)	−0.0057 (16)	0.0098 (16)	−0.0195 (17)
C27	0.020 (3)	0.024 (3)	0.024 (3)	0.002 (2)	0.010 (2)	0.001 (2)
C26	0.016 (2)	0.022 (3)	0.017 (3)	0.002 (2)	0.009 (2)	0.001 (2)
C21	0.018 (3)	0.017 (2)	0.022 (3)	−0.003 (2)	0.009 (2)	−0.002 (2)
C25	0.012 (2)	0.017 (2)	0.017 (2)	−0.0018 (19)	0.0051 (19)	0.0003 (19)
Mg3	0.0226 (9)	0.0199 (9)	0.0195 (9)	−0.0003 (7)	0.0119 (7)	−0.0010 (7)
O51	0.037 (2)	0.031 (2)	0.025 (2)	−0.0034 (16)	0.0099 (18)	0.0031 (16)
O61	0.070 (3)	0.048 (3)	0.046 (3)	0.006 (2)	0.008 (3)	0.016 (2)
N51	0.046 (3)	0.037 (3)	0.033 (3)	0.004 (2)	0.014 (2)	0.013 (2)
N61	0.061 (4)	0.046 (3)	0.035 (3)	0.022 (3)	0.012 (3)	0.020 (3)
C51	0.041 (3)	0.018 (3)	0.049 (4)	−0.005 (2)	0.027 (3)	−0.002 (3)
C52	0.079 (5)	0.040 (4)	0.072 (5)	−0.010 (4)	0.021 (4)	0.020 (4)
C61	0.076 (6)	0.052 (5)	0.065 (5)	−0.001 (4)	0.017 (5)	−0.018 (4)
C22	0.016 (3)	0.019 (3)	0.021 (3)	−0.0042 (19)	0.005 (2)	0.000 (2)
C53	0.078 (5)	0.077 (5)	0.040 (4)	0.015 (4)	0.008 (4)	0.016 (4)
C28	0.017 (3)	0.017 (3)	0.017 (3)	0.001 (2)	0.007 (2)	0.002 (2)
C24	0.022 (3)	0.024 (3)	0.013 (3)	0.004 (2)	0.010 (2)	0.005 (2)
C23	0.013 (2)	0.023 (3)	0.018 (3)	0.0023 (19)	0.007 (2)	0.005 (2)
C62	0.119 (8)	0.101 (8)	0.149 (10)	−0.015 (6)	0.093 (8)	−0.014 (7)
C63	0.084 (7)	0.117 (8)	0.126 (9)	0.046 (6)	0.007 (6)	0.001 (7)
O23	0.0175 (17)	0.0287 (19)	0.0167 (19)	0.0030 (14)	0.0034 (14)	−0.0011 (15)
O24	0.0172 (18)	0.0272 (19)	0.0193 (18)	0.0035 (14)	0.0082 (14)	−0.0009 (15)
O26	0.0153 (18)	0.035 (2)	0.0196 (19)	−0.0070 (15)	0.0069 (14)	−0.0042 (15)
C29	0.017 (3)	0.025 (3)	0.019 (3)	0.004 (2)	0.009 (2)	0.000 (2)
O25	0.0253 (19)	0.038 (2)	0.0190 (19)	0.0034 (16)	0.0132 (15)	0.0083 (16)
C43	0.148 (9)	0.093 (7)	0.090 (7)	−0.018 (7)	0.042 (7)	−0.055 (6)

### Geometric parameters (Å, º)

**Table:**

Mg1—O14^i^	1.997 (3)	C33—H33B	0.9600
Mg1—O24^ii^	2.041 (3)	C33—H33C	0.9600
Mg1—O15^iii^	2.072 (3)	O22—C27	1.256 (5)
Mg1—O12	2.169 (3)	Mg3—O22	2.104 (4)
Mg1—O11	2.202 (3)	C27—C21	1.500 (6)
Mg1—O21	2.233 (3)	C27—Mg3	2.526 (5)
Mg1—C17	2.523 (5)	C26—C25	1.387 (6)
Mg2—O23^ii^	2.019 (3)	C26—C21	1.398 (6)
Mg2—O16^iii^	2.023 (4)	C26—H26	0.9300
Mg2—O12	2.058 (3)	C21—C22	1.376 (6)
Mg2—O41	2.081 (4)	C25—C24	1.388 (6)
Mg2—O31	2.091 (4)	C25—C29	1.512 (6)
Mg2—O26	2.113 (4)	Mg3—O13^i^	1.990 (3)
O12—C17	1.279 (5)	Mg3—O25^i^	2.024 (3)
O14—C18	1.252 (5)	Mg3—O51	2.089 (4)
O14—Mg1^iv^	1.997 (3)	Mg3—O61	2.110 (5)
O11—C17	1.245 (5)	O51—C51	1.234 (6)
O15—C19	1.262 (5)	O61—C61	1.160 (8)
O15—Mg1^v^	2.072 (3)	N51—C51	1.320 (6)
O13—C18	1.256 (5)	N51—C52	1.446 (7)
O13—Mg3^iv^	1.990 (3)	N51—C53	1.448 (7)
O16—C19	1.249 (5)	N61—C61	1.330 (9)
O16—Mg2^v^	2.023 (4)	N61—C62	1.411 (9)
C13—C12	1.383 (6)	N61—C63	1.417 (9)
C13—C14	1.399 (6)	C51—H51	0.9300
C13—C18	1.504 (6)	C52—H52A	0.9600
C12—C11	1.389 (6)	C52—H52B	0.9600
C12—H12	0.9300	C52—H52C	0.9600
C11—C16	1.380 (6)	C61—H61	0.9300
C11—C17	1.493 (6)	C22—C23	1.395 (6)
C14—C15	1.393 (6)	C22—H22	0.9300
C14—H14	0.9300	C53—H53A	0.9600
C16—C15	1.390 (6)	C53—H53B	0.9600
C16—H16	0.9300	C53—H53C	0.9600
C15—C19	1.496 (6)	C28—O23	1.242 (5)
O31—C31	1.223 (6)	C28—O24	1.260 (5)
O41—C41	1.213 (7)	C28—C23	1.513 (6)
C41—N41	1.316 (7)	C24—C23	1.386 (6)
C41—H41	0.9300	C24—H24	0.9300
N41—C43	1.452 (10)	C62—H62A	0.9600
N41—C42	1.468 (9)	C62—H62B	0.9600
C42—H42A	0.9600	C62—H62C	0.9600
C42—H42B	0.9600	C63—H63A	0.9600
C42—H42C	0.9600	C63—H63B	0.9600
O21—C27	1.275 (5)	C63—H63C	0.9600
Mg3—O21	2.279 (3)	O23—Mg2^ii^	2.019 (3)
N31—C31	1.322 (7)	O24—Mg1^ii^	2.041 (3)
N31—C33	1.458 (7)	O26—C29	1.249 (5)
N31—C32	1.461 (8)	C29—O25	1.267 (5)
C31—H31	0.9300	O25—Mg3^iv^	2.024 (3)
C32—H32A	0.9600	C43—H43A	0.9600
C32—H32B	0.9600	C43—H43B	0.9600
C32—H32C	0.9600	C43—H43C	0.9600
C33—H33A	0.9600		
			
O14^i^—Mg1—O24^ii^	109.27 (14)	H33A—C33—H33B	109.5
O14^i^—Mg1—O15^iii^	85.72 (14)	N31—C33—H33C	109.5
O24^ii^—Mg1—O15^iii^	99.80 (14)	H33A—C33—H33C	109.5
O14^i^—Mg1—O12	151.66 (14)	H33B—C33—H33C	109.5
O24^ii^—Mg1—O12	99.06 (13)	C27—O22—Mg3	94.1 (3)
O15^iii^—Mg1—O12	88.64 (13)	O22—C27—O21	120.2 (4)
O14^i^—Mg1—O11	92.30 (13)	O22—C27—C21	119.1 (4)
O24^ii^—Mg1—O11	156.35 (15)	O21—C27—C21	120.7 (4)
O15^iii^—Mg1—O11	91.13 (14)	O22—C27—Mg3	56.2 (2)
O12—Mg1—O11	60.03 (12)	O21—C27—Mg3	64.1 (2)
O14^i^—Mg1—O21	94.47 (13)	C21—C27—Mg3	174.9 (3)
O24^ii^—Mg1—O21	83.25 (13)	C25—C26—C21	120.6 (4)
O15^iii^—Mg1—O21	176.71 (15)	C25—C26—H26	119.7
O12—Mg1—O21	89.64 (12)	C21—C26—H26	119.7
O11—Mg1—O21	85.58 (13)	C22—C21—C26	119.2 (4)
O14^i^—Mg1—C17	121.63 (15)	C22—C21—C27	120.7 (4)
O24^ii^—Mg1—C17	128.79 (15)	C26—C21—C27	120.0 (4)
O15^iii^—Mg1—C17	89.68 (15)	C26—C25—C24	119.0 (4)
O12—Mg1—C17	30.46 (13)	C26—C25—C29	118.2 (4)
O11—Mg1—C17	29.57 (13)	C24—C25—C29	122.8 (4)
O21—Mg1—C17	87.42 (14)	O13^i^—Mg3—O25^i^	106.92 (15)
O23^ii^—Mg2—O16^iii^	93.05 (15)	O13^i^—Mg3—O51	92.30 (15)
O23^ii^—Mg2—O12	91.71 (13)	O25^i^—Mg3—O51	94.69 (15)
O16^iii^—Mg2—O12	87.33 (14)	O13^i^—Mg3—O22	149.56 (15)
O23^ii^—Mg2—O41	89.05 (15)	O25^i^—Mg3—O22	102.80 (14)
O16^iii^—Mg2—O41	89.75 (15)	O51—Mg3—O22	91.66 (15)
O12—Mg2—O41	177.03 (16)	O13^i^—Mg3—O61	85.01 (18)
O23^ii^—Mg2—O31	172.81 (16)	O25^i^—Mg3—O61	86.15 (17)
O16^iii^—Mg2—O31	88.69 (15)	O51—Mg3—O61	177.30 (18)
O12—Mg2—O31	95.34 (14)	O22—Mg3—O61	90.65 (18)
O41—Mg2—O31	83.98 (15)	O13^i^—Mg3—O21	90.22 (13)
O23^ii^—Mg2—O26	90.85 (13)	O25^i^—Mg3—O21	162.75 (14)
O16^iii^—Mg2—O26	175.89 (14)	O51—Mg3—O21	86.36 (14)
O12—Mg2—O26	93.85 (14)	O22—Mg3—O21	59.94 (12)
O41—Mg2—O26	89.01 (15)	O61—Mg3—O21	93.61 (16)
O31—Mg2—O26	87.28 (14)	O13^i^—Mg3—C27	120.26 (16)
C17—O12—Mg2	142.9 (3)	O25^i^—Mg3—C27	132.54 (16)
C17—O12—Mg1	90.2 (3)	O51—Mg3—C27	88.59 (15)
Mg2—O12—Mg1	114.79 (14)	O22—Mg3—C27	29.74 (13)
C18—O14—Mg1^iv^	134.8 (3)	O61—Mg3—C27	92.74 (18)
C17—O11—Mg1	89.7 (3)	O21—Mg3—C27	30.21 (13)
C19—O15—Mg1^v^	129.2 (3)	C51—O51—Mg3	125.4 (3)
C18—O13—Mg3^iv^	130.3 (3)	C61—O61—Mg3	135.6 (5)
C19—O16—Mg2^v^	138.7 (3)	C51—N51—C52	121.0 (5)
C12—C13—C14	119.5 (4)	C51—N51—C53	122.3 (5)
C12—C13—C18	117.5 (4)	C52—N51—C53	116.7 (5)
C14—C13—C18	123.0 (4)	C61—N61—C62	123.1 (7)
C13—C12—C11	120.8 (4)	C61—N61—C63	116.7 (7)
C13—C12—H12	119.6	C62—N61—C63	120.1 (7)
C11—C12—H12	119.6	O51—C51—N51	124.4 (5)
C16—C11—C12	118.8 (4)	O51—C51—H51	117.8
C16—C11—C17	121.1 (4)	N51—C51—H51	117.8
C12—C11—C17	120.1 (4)	N51—C52—H52A	109.5
C15—C14—C13	120.3 (4)	N51—C52—H52B	109.5
C15—C14—H14	119.9	H52A—C52—H52B	109.5
C13—C14—H14	119.9	N51—C52—H52C	109.5
O11—C17—O12	120.1 (4)	H52A—C52—H52C	109.5
O11—C17—C11	120.5 (4)	H52B—C52—H52C	109.5
O12—C17—C11	119.2 (4)	O61—C61—N61	129.8 (8)
O11—C17—Mg1	60.8 (2)	O61—C61—H61	115.1
O12—C17—Mg1	59.3 (2)	N61—C61—H61	115.1
C11—C17—Mg1	176.7 (3)	C21—C22—C23	121.3 (4)
O14—C18—O13	125.9 (4)	C21—C22—H22	119.4
O14—C18—C13	117.6 (4)	C23—C22—H22	119.4
O13—C18—C13	116.5 (4)	N51—C53—H53A	109.5
C11—C16—C15	121.8 (4)	N51—C53—H53B	109.5
C11—C16—H16	119.1	H53A—C53—H53B	109.5
C15—C16—H16	119.1	N51—C53—H53C	109.5
C16—C15—C14	118.5 (4)	H53A—C53—H53C	109.5
C16—C15—C19	117.7 (4)	H53B—C53—H53C	109.5
C14—C15—C19	123.7 (4)	O23—C28—O24	125.8 (4)
O16—C19—O15	125.5 (4)	O23—C28—C23	116.1 (4)
O16—C19—C15	115.9 (4)	O24—C28—C23	118.0 (4)
O15—C19—C15	118.6 (4)	C23—C24—C25	121.5 (4)
C31—O31—Mg2	122.5 (4)	C23—C24—H24	119.3
C41—O41—Mg2	125.3 (4)	C25—C24—H24	119.3
O41—C41—N41	126.4 (6)	C24—C23—C22	118.4 (4)
O41—C41—H41	116.8	C24—C23—C28	123.0 (4)
N41—C41—H41	116.8	C22—C23—C28	118.4 (4)
C41—N41—C43	119.1 (7)	N61—C62—H62A	109.5
C41—N41—C42	120.8 (7)	N61—C62—H62B	109.5
C43—N41—C42	119.9 (7)	H62A—C62—H62B	109.5
N41—C42—H42A	109.5	N61—C62—H62C	109.5
N41—C42—H42B	109.5	H62A—C62—H62C	109.5
H42A—C42—H42B	109.5	H62B—C62—H62C	109.5
N41—C42—H42C	109.5	N61—C63—H63A	109.5
H42A—C42—H42C	109.5	N61—C63—H63B	109.5
H42B—C42—H42C	109.5	H63A—C63—H63B	109.5
C27—O21—Mg1	141.7 (3)	N61—C63—H63C	109.5
C27—O21—Mg3	85.7 (3)	H63A—C63—H63C	109.5
Mg1—O21—Mg3	117.68 (15)	H63B—C63—H63C	109.5
C31—N31—C33	120.8 (5)	C28—O23—Mg2^ii^	142.6 (3)
C31—N31—C32	122.0 (5)	C28—O24—Mg1^ii^	127.5 (3)
C33—N31—C32	116.9 (5)	C29—O26—Mg2	148.8 (3)
O31—C31—N31	123.8 (5)	O26—C29—O25	124.1 (4)
O31—C31—H31	118.1	O26—C29—C25	117.9 (4)
N31—C31—H31	118.1	O25—C29—C25	118.0 (4)
N31—C32—H32A	109.5	C29—O25—Mg3^iv^	129.0 (3)
N31—C32—H32B	109.5	N41—C43—H43A	109.5
H32A—C32—H32B	109.5	N41—C43—H43B	109.5
N31—C32—H32C	109.5	H43A—C43—H43B	109.5
H32A—C32—H32C	109.5	N41—C43—H43C	109.5
H32B—C32—H32C	109.5	H43A—C43—H43C	109.5
N31—C33—H33A	109.5	H43B—C43—H43C	109.5
N31—C33—H33B	109.5		

Symmetry codes: (i) *x*, −*y*+1/2, *z*−1/2; (ii) −*x*, −*y*+1, −*z*; (iii) −*x*+1, *y*+1/2, −*z*+1/2; (iv) *x*, −*y*+1/2, *z*+1/2; (v) −*x*+1, *y*−1/2, −*z*+1/2.
